# Thoracic petechiae in an adult woman

**DOI:** 10.1016/j.jdcr.2026.02.041

**Published:** 2026-02-28

**Authors:** Felix Bui, Noor Hasan, Sandra Morris, Joseph M. Dyer

**Affiliations:** a Philadelphia College of Osteopathic Medicine, Georgia Campus, Suwanee, Georgia; b Epiphany Dermatology, Peachtree City, Georgia

**Keywords:** amyloidosis, cutaneous amyloid, petechiae, purpura

## Case

A 58-year-old female patient presented with nonpruritic petechiae and purpuric macules scattered on the upper chest for several months. The patient denied fevers, joint aches, weight loss, fatigue, estrogen replacement, or nonsteroidal anti-inflammatory drug use. Serum vitamin C, vitamin K, complete blood count, coagulation studies, and comprehensive metabolic panel were all unremarkable.

**Fig 1:**
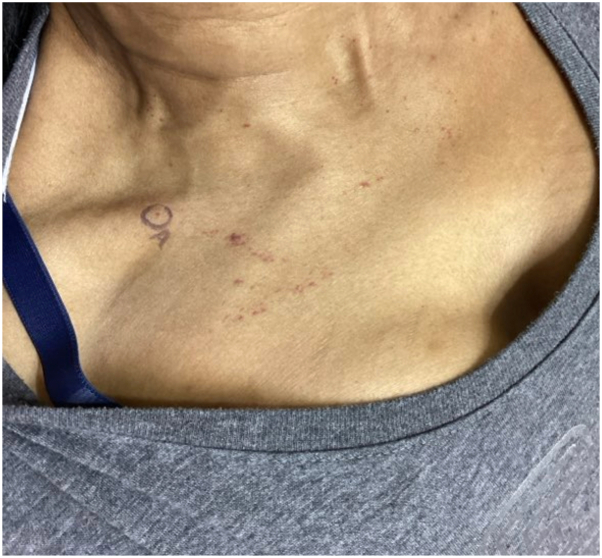
Few petechiae of the upper thorax. Biopsy site circled on right clavicular skin.

**Fig 2:**
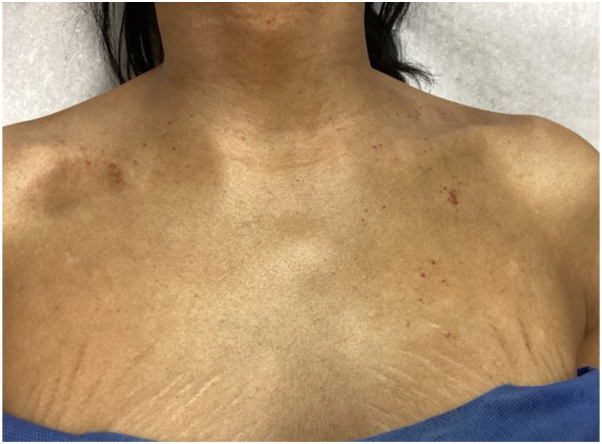
Few petechiae and purpuric macules on the chest, 6 weeks after biopsy.

**Fig 3:**
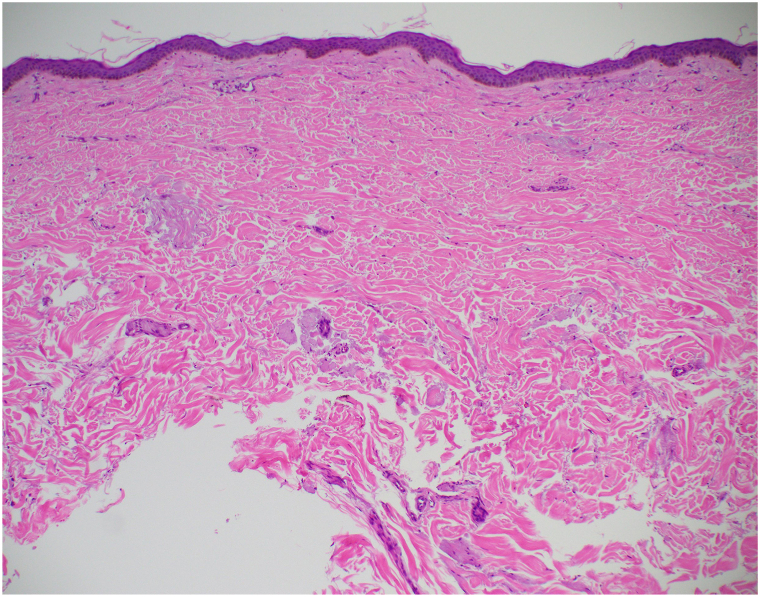
Wispy lavender-gray amorphous deposits are visible throughout the dermis (H&E, original magnification 4×). *H&E*, Hematoxylin and Eosin staining.

**Fig 4:**
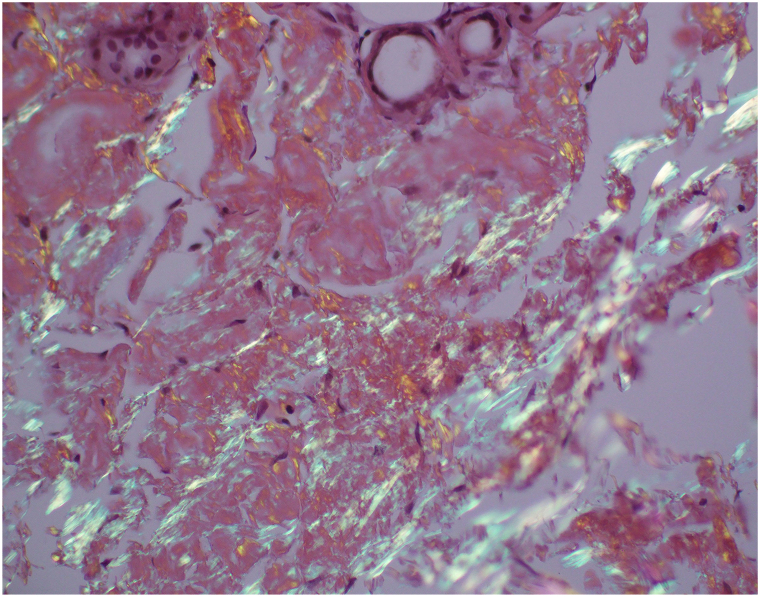
Amyloid deposits in the dermis of a skin punch biopsy showing apple-green birefringence under polarized light (Congo red stain, original magnification 20×).

One year later, the patient returned to the dermatology clinic for re-evaluation as the petechiae remained unresolved and were increasing in number (Figs 1 and 2). Systemic symptoms were absent, aside from complaint of chest pain on exertion. Punch biopsy revealed amorphous hyaline deposits through the full thickness of the dermis and into the subcutaneous tissue on Hematoxylin & Eosin staining (Fig 3); these deposits demonstrated apple-green birefringence under polarized light and Congo red stain (Fig 4). Immunohistochemical staining was negative for keratin amyloid. Serum protein electrophoresis exhibited a discrete abnormal band. Bone marrow biopsy demonstrated a preponderance of plasma cells greater than 40%.

### Question: What is the most likely diagnosis?

**A.** Amyloid A protein (AA) amyloidosis
**B.** Amyloid light chain amyloidosis
**C.** Lichen amyloidosis (LA)
**D.** Macular amyloidosis (MA)
**E.** Nodular amyloidosis (NA)

## Answer and discussion

Correct answer: B. Amyloid light chain amyloidosis.

Amyloid light chain amyloidosis (ALA) is a systemic disease caused by misfolded immunoglobulin light chains produced by a monoclonal plasma cell dyscrasia (eg, monoclonal gammopathy of undetermined significance, Waldenstrom’s macroglobulinemia, or multiple myeloma). These systemically produced light chains deposit as amyloid in various tissues, such as heart, kidneys, nerves, liver, gastrointestinal tract, and skin.^1^

Primary cutaneous amyloidosis includes LA, MA, and NA. LA and MA result from degenerated local keratin, making those answer choices incorrect as our patient’s amyloid was negative for a keratin source by immunohistochemistry. NA results from light chains produced by local plasma cells, not a monoclonal population of plasma cells, as evidenced by our patient’s serum protein electrophoresis and bone marrow biopsy.

It should also be noted that our case may be clinically delineated from MA, LA, and NA. MA is typically hyperpigmented, not purpuric, and itching may be prominent. LA commonly presents as hyperpigmented plaques, not purpuric patches, which are significantly lichenified due to pruritus. Finally, NA presents with apparent nodules, not petechiae.

Systemic amyloidosis includes AA amyloidosis, ALA, and others. AA amyloidosis occurs after a hepatogenic acute phase reactant, serum AA protein deposits in tissue; it is associated with rheumatoid arthritis, inflammatory bowel disease, chronic infection, or malignancy.^1^ Our patient’s monoclonal gammopathy makes light chain–derived amyloid far more likely than acute phase reactant–derived amyloid.

ALA presents classically with periorbital (so-called “pinch”) purpura, waxy papulonodules, macroglossia, ecchymoses, and deltoid hypertrophy. The combination of periorbital purpura and macroglossia is pathognomonic for ALA, occurring in about 15% of patients.^2^ A 2019 retrospective study from Mexico reported on 48 patients with ALA, 2 of which demonstrated purpuric macules on the torso, as in our patient, although it was not specified whether these 2 patients exhibited additional skin or mucosa findings as well.^3^ Less commonly reported findings of ALA include genital ulcers, xerostomia, and anonychia.^3^ With other stereotypical and noteworthy mucocutaneous findings conspicuously absent from our patient’s case, thoracic petechiae and purpuric macules were left as the sole (fairly subtle) clue to her diagnosis.

## Conflicts of interest

None disclosed.
